# Laparoscopic cholecystectomy in sickle cell patients in Niger

**Published:** 2009-12-06

**Authors:** Sani Rachid, Lassey James Didier, Mallam Abdou Badé, Chaibou Maman Sani, Abarchi Habibou

**Affiliations:** 1 Department of general and digestive surgery, National Hospital of Niamey PB: 238 Niamey- Niger,; 2 Department of haematology and oncology, National Hospital of Niamey PB: 238 Niamey- Niger,; 3 Department of anaesthesiology, National Hospital of Niamey PB: 238 Niamey- Niger,; 4 Department of paediatric surgery, National Hospital of Niamey PB: 238 Niamey- Niger

**Keywords:** Sickle cell disease, laparoscopic cholecystectomy, Niger

## Abstract

**Background:**

We report the results of our experience on laparoscopic cholecystectomy in sickle cell disease patients in Niger, which is included in the sickle cell belt.

**Methods:**

A prospective study covering a period of 45 months, from July 2004 to March 2008. We included all sickle cell disease patients that underwent laparoscopic cholecystectomy. Blood transfusion was done for patients with haemoglobin (Hb) levels less than 9g/dl. Homozygous and composite heterozygous patients were admitted in intensive care unit for 24 hours or plus post operatively.

**Results:**

The series included 47 patients operated by the same surgeon, 31 females (66%) and 16 males (34%) (Ratio: 0.51). The average age was 22.4 years (range: 11 to 46 years) and eleven (23.4%) of them were aged less than 15 years. The types of sickle cell disease found were 37 SS, 2 SC, 1 S beta-thalassemia and 7 AS. Indications for surgery were biliary colic in 29 cases (61.7%) and acute cholecystitis in 18 cases (38.3%). The mean operative time was 64 min (range: 42 to 103 min). Conversion to open cholecystectomy in 2 cases (4.2 %) for non recognition of Calot‘s triangle structures. The postoperative complications were: four (4) cases of vaso-occlusive crisis and one case of acute chest syndrome. The mean postoperative hospital stay was 3,5days (range: 1 to 9 days). No mortality was encountered.

**Conclusion:**

Laparoscopic cholecystectomy is a safe procedure in sickle cell patients. It should be a multidisciplinary approach and involve a haematologist, an anaesthesiologist and a surgeon.

## Background

Sickle cell disease is one of the most common haemoglobinopathies in Africa; it is a serious community health problem in some sub-saharan countries. Its prevalence is varies from 18 to 25% in Niger. Gallstone is a major abdominal complication of chronic haemolysis. General anaesthesia and cholecystectomy can be carried out on sickle cell patients with a very high risks of complications (vaso occlusive crisis and acute chest syndrome). Laparoscopic cholecystectomy (LC) requires expert anaesthesia and a well trained surgeon. Sickling is avoided by preventing hypoxia, hypovolaemia, hypothermia and acidosis; LC is recommended by many authors because of its safety and with a short period of hospital stay [1–5]. The aim of this study is to report our experience on laparoscopic cholecystectomy in sickle cell patients in Niger.

## Method

It’s a prospective study covering a period of 45 months; from July 2004 to March 2008. Were included in the study, sickle cell patients with simple or complicated gallstones admitted in general and digestive surgery unit of the National Hospital of Niamey. Patients with contraindications to pneumoperitonium and those with common bile duct stones were excluded. The preoperative investigations include a hepatic function test, amylasemia; malaria test and ultrasound scan of the hepatobiliary tree. A cardiac echography and lung function test were done depending on the findings on clinical examination. The preoperative management was conducted by the anaesthesiologist and includes hydration, antibiotics (3 ^rd^ generation cephalosporin and metronidazole ); blood transfusion for haemoglobin (Hb) levels below 9g/dl. Postoperatively homozygous and composite heterozygous patients were admitted into the intensive care unit for special reanimation during the first 24 hours or more. The American method was used for the surgical operation. The position of trocars depends mainly on the difficulty of the dissection (past history of abdominal surgery). Our study reviewed the following parameters: age and sex, indication and duration of operation, type of intraoperative incidents and conversion to laparotomy, post operative complications and duration of post operative hospital stay.

## Results

From July 2004 to March 2008, 127 LC were performed in the digestive surgery unit of the National Hospital of Niamey. 47 cases of LC (37%) were done on SCD patients with 16 males (34%) and 31 females (66%) (sex.ratio: 0.51). The mean age was 22.4 years (range 11 to 46 years); eleven patients (23.4%) were aged less than 15 years, of whom 7 patients (14.9%) had a past history of surgical operation under general anaesthesia. Haemoglobin electrophoresis showed 37 cases (78.7%) of homozygous HbSS, seven heterozygous (14.9%) Hb AS, 3 composite heterozygous: two cases (4.2%) of HbSC and one case of HbS-beta thalassemia (2.1%). Indications for surgery were biliary colic in 29 cases (61.7%) and acute cholecystitis in 18 cases (38.3%). Preoperatively 27 patients (57.4%) with Hb levels less than 9g/dl were transfused; 7 patients (14.9%) with positive malaria tests were treated with antimalarial drugs ( Table 1 ).

The operations were carried out by the same surgeon. Injuries reported during this procedure included accidental gallbladder perforation in 6 cases (12.8%) and haemorrhage in one case (2.1%). Total cholecystectomy was performed in 44 cases (93.6%) and partial cholecystectomy, leaving a small portion of gallbladder tissue adhered to the liver was performed 3 cases (6.4%). The mean operation time was 64 minutes (range 42 to 103 minutes); the mean anaesthesia time was 96 minutes (range 85 to 157 minutes). Conversion to laparotomy happened in 2 cases (4.2%) due to non recognition of the structures in the Calot’s triangle caused by inflammation and haemorrhage. Drainage was performed in 2 cases (4.2%). Forty patients (85.1%) were follow up in the intensive care unit postoperatively as follows: 35 patients (32 HbSS, 2 HbSC, 1 HbS beta-thalassemia) stayed for the first 24hrs post operatively; 4 patients HbSS (8.5%) stayed for 3 days because of vaso-occlusive crisis (VOC), 1 patient HbSS (2.1%) had acute chest syndrome (ACS) and stayed for 7days under oxygen therapy and antibiotics.

The total of these complications were amounted 5 (4 VOC and 1 ACS) representing 10.6% of all LC performed on SCD patients. VOC and ACS occurred in non transfused patients preoperatively. For postoperative analgesia, 10 patients (21.3%) received nalbuphin (morphinic derivative) and the others (78.7%) received intra-venous paracetamol. Feeding was started post operatively at day 0 to day1 for 40 patients (85.1%); day 2 for 4 patients (8.5%) and day 3 for 3 patients (6.4%). The mean post operative length of stay in hospital 3.5 days (range: 1 to 9 days). One patient (2.1%) was seen for follow up on day 12 post-op with complains of fever; an ultrasound scan showed a subhepatic abscess subsequently treated with antibiotics (ceftriaxone and metronidazole). In total, post operative complications were estimated at 12.8% and no mortality occurred.

## Discussion

Gallstones are a frequent complication in patients with sickle cell disease (SCD) because of the recurrent episodes of haemolysis leading to an increase in bilirubin excretion and pigment gallstones formation. The incidence of gallstones in patients with SCD increased in these last years due to both the regular use of the non-invasive detection technique (ultrasonography), best follow-up care for patients, and the longer survival of these patients. The prevalence of cholelithiasis in patients with SCD varies between countries. It is estimated at 34 to 70% in USA, 4 to 25% in Africa and 8% in Saudi Arabia [1]. In our country, more than one third of patient who underwent laparoscopic cholecystectomy had SCD (37%). The same preavalence was found in Senegal [1].

The management of SCD is multidisciplinary and involves the anaesthesiologist, the haematologist and the surgeon. Together they assess the patient’s fitness for surgery. Intensive care unit stay postoperatively is recommended by many authors [1–6] for specific reanimation preventing VOC and ACS (oxygen therapy, hydratation, antibiotics monitoring with pulse oximetry). Transfusion of red cells reduces the proportion of sickle erythrocytes and correct anaemia. Its modalities (simple and exchanged blood transfusion) have been debated by many authors ,Vichinsky and al [6] in a multicenter study which patients were randomly assigned to undergo an aggressive regimen of transfusions designed to maintain a preoperative hemoglobin level of 10 g/dl (range 9 to 11) and a hemoglobin S level of 30 percent or less (group 1) or a conservative transfusion regimen designed to maintain the hemoglobin level at 10 g/dl (range 9 to 11), regardless of the percentage of hemoglobin S (group 2); the results showed that the incidence of postoperative specific complications were the same in the two groups (10% of ACS and VOC) and the blood transfusion complications were higher in the group with aggressive transfusion (40% vs 20%). In our study, blood transfusion is indicated in patients with Hb levels inferior to 9g/dl. Meshikhes and al [7] showed that blood transfusion in patients with Hb less than 10g/dl can prevent hypoxia and other complications leading to sickle cell. Prevention of hyperthermia and anti malarial prophylactic was essential in the management of sickle cell disease. Malaria is endemic in our country, Djibo and al [9] in a study in 2001 showed that antimalarial prophylaxis in patients with positive malaria test is very important because of its preventive effect in surgical patients, and in the SCD it’s often post-transfusional malaria.

The mean duration time for LC is 64 minutes (range 42 to 103 minutes) in our study. Fall and others [1] found 60 minutes (range 30 to 90min) ( Table 2 ). Leandros and al [4] who compared laparoscopy and laparotomy found that the mean operation time was 81.4 minutes (range: 55–125 min) for open cholecystectomy and 64.2 min (range: 45–90 min) for laparoscopic cholecystectomy (p < 0.01). Complications occurred in 5% (2/41) of patients in the laparoscopic group and in 20% (8/41) of patients in the laparotomy group (p = 0.04). The mean length of stay in hospital was 5.6 days (range 3 to 9 days) in the open group and 2.7 days (range 2 to 5 days) in the laparoscopic group (p < 0.01). For many authors laparoscopic cholecystectomy is the gold standard for gallstone in SCD [1–5, 7, 11–13]. Conversion to open operation was necessary in 2 patients (4.2%) in our series, due to haemorrhage and non recognition of structures of the Calot’s triangle. In the literature the main causes of conversion to laparotomy are: bleeding, common bile duct injuries and difficult apprehension of gall bladder especially in chronically inflamed cases. The conversion to open cholecystectomy is a way out in case of intraoperative complications. In our study none of these life-threatening complications occurred in transfused patients. In Haberkern and al [13] series, the rate of these specifics complications was 39% and their study showed that high risk of post operative complications are more common in preoperatively non transfused patients. The laparoscopic cholecystectomy is associated with low mortality and morbidity ( Table 2 ).

In our study only symptomatic gallstones were operated, but in the future, the surgical management of SCD patients will also handle non symptomatic gallstones. Curro and al [14] compared two groups of patients with asymptomatic gallstones. Group A patients have elective LC and group B refused the surgery, this study recommended that LC is highly beneficial because it prevented complications (acute cholecystitis, common bile duct stones and hepatic colitis) and periodic hospitalization and all the burden associated with it. The correlation between cholecystectomy performed in asymptomatic children (group A) and cholecystectomy performed in symptomatic children (group B) showed siqgnificant differences in the outcome. Morbidity rate and postoperative stay increased when children with SCD underwent emergency LC. LC is a safe procedure in sickle cell patients. There was only one case of acute chest syndrome in this study and it affected the right lung. For Crawford and al, ACS complicating cholecystectomy or splenectomy shows a predilection for basal lung regions on the side of surgery. Patients were more likely to have new infiltrates involving the lung on the side of the surgery or bilateral than isolated controlateral side (P < 0.0001) [15].

## Conclusion

In our environment, LC is a safe procedure in sickle cell patients with a very low morbidity and mortality rate .It’ s a multidisciplinary approach involving a hematologist, an anesthesiologist and a surgeon. The factors in predisposing to vaso occlusive crisis should be monitored during the pre, intra and post operative management.

## Figures and Tables

**Table 1:** Preoperative management

** Pre-operative treatment **	** Percentage **
Hydratation	68.1% (n=32)
Transfusion	57.4% (n=27)
Antibiotics	40.4% (n=19)
Anti malarial treatment	14.9% (n=7)

**Table 2:** Results of LC in sickle cell patients: some series compared to ours

** Authors **	** Years **	** Numbers of patients **	** Operating time (mean, range) **	** Conversion rate % **	** Complications % **	** Mortality % **
Meshikles [7]	1995	30	60–100 min	3,3	6.6	3.3
Al- Abkhari [11]	2001	36	-	0	7.7	0
Al Mulhim [12]	2002	35	-	5.7	17.5	0
Fall and al [1]	2003	42	60 min	1.7	16.7	1.7
Plummer [10]	2006	16	70– 150 min	25	37.5	6.25
** Our study **	2008	47	64 min	4.2	12.7	0
